# The Effect of Patient-Provider Relationships on Compliance in Uncontrolled Diabetic Patients

**DOI:** 10.7759/cureus.84910

**Published:** 2025-05-27

**Authors:** Victoria Comfort, Erin N Burns, Alexis K Grover, Lillian A Reed-Harrington, Danielle Fastring

**Affiliations:** 1 College of Osteopathic Medicine, William Carey University, Hattiesburg, USA

**Keywords:** communication, compliance, diabetes, patient-provider relationship, uncontrolled diabetes

## Abstract

Background

Effective patient-provider communication is critical for managing chronic diseases like diabetes mellitus (DM). This study examines whether patient attitudes toward their healthcare providers influence diabetes management and compliance, comparing individuals with poorly managed diabetes mellitus (PM DM) and those without DM (No DM).

Methods

Data from the All of Us Research Program was analyzed. After excluding incomplete responses, the final sample included 1903 PM DM patients and 94,218 No DM patients. Patient perceptions of respect, involvement in medical decision making, and provider interactions were assessed. Bivariate analysis was used to identify responses that were independently associated with PM DM. Variables found to be independently associated with PM DM at p<0.10 were entered into a multivariable model, and backwards stepwise binary logistic regression was used to find the model of best fit. Variables found to be significant (p<0.05) in the multivariable model were retained.

Results

Patients who felt disrespected by providers were significantly more likely to have poorly managed diabetes (OR = 1.396, 95% CI: 1.189, 1.639, p<0.05). Counterintuitively, patients who reported always being asked for their opinion in treatment decisions were likely to have PM DM (OR = 1.466, 95% CI: 1.235, 1.740, p<0.05). Patients who were not nervous to see their provider were also more likely to have PM DM (OR = 1.365, 95% CI: 1.152, 1.618, p<0.05).

Conclusion

Findings suggest that positive provider interaction does not always translate to better disease control. Future studies should explore how communication styles and shared decision-making impact long-term adherence to diabetes treatment.

## Introduction

Patient adherence and compliance with medical treatment play a critical role in health outcomes, particularly for chronic diseases. Adherence has been described as "an active process in which a patient takes responsibility for their overall wellbeing", while compliance has been described as "a passive behavior in which a patient is following a list of recommendations from the doctor" [1,2]. While these concepts are distinct, both are influenced by multiple factors, including patient-provider relationships [3]. 

The quality of communication between a patient and provider is essential for compliance. Effective communication involves gathering patient history, diagnosing conditions, creating treatment plans, and providing ongoing support [4]. Trust, respect, and shared decision-making have been identified as key components of a healthy physician-patient relationship [5,6]. A meta-analysis of 127 studies found that patients whose physicians communicate poorly are 19% more likely to be non-adherent [7]. Given this strong link, understanding how provider interactions affect patient compliance is essential for improving health outcomes [8].

Beyond communication, sociocultural factors also shape the patient-provider dynamic. Race, ethnicity, religion, and language influence how patients perceive and engage with healthcare. Research shows that Hispanic patients are 19 times more likely to choose a Hispanic doctor as their regular doctor than a non-minority patient is [9]. Furthermore, patients report receiving higher quality care and being more involved in their own treatment plan when their doctor shares their ethnic background [10]. Minority patients may seek care from physicians who align with their race and ethnicity because of personal preference and language, and not solely based on geographic accessibility [11]. Patients who are of the same race, ethnicity, and religion as their doctors often report that it is easier to communicate with their providers [11]. These findings suggest that cultural alignment between patients and providers may improve engagement in disease management. 

Diabetes mellitus (DM) is a chronic disease requiring long-term adherence to medication, lifestyle changes, and routine medical care. According to the Centers for Disease Control and Prevention (CDC), as of 2021, 38.4 million Americans have diabetes [12], making it a leading public health concern. However, diabetes ranks second among 17 chronic diseases in terms of poor treatment adherence, contributing significantly to hospitalizations due to non-compliance [13]. Studies suggest that patient-provider interactions are critical to diabetes adherence. An analysis of primary care physicians at Geisinger Clinic found that provider communication and patient beliefs about medication necessity were significant predictors of adherence [14]. Similarly, a qualitative study exploring barriers to medication adherence in patients with type 2 diabetes found that lack of trust in physicians and inconsistent provider recommendations were major contributors to non-compliance [15]. Another qualitative study explored how the patient-provider relationship impacted initial insulin acceptance and ongoing compliance, identifying trust, effective communication, patient-centered decision-making, and continuity of care as factors that positively influence both [16]. However, while prior studies have examined these factors in smaller clinical settings, there is a lack of large-scale, diverse datasets evaluating the relationship between patient-provider dynamics and diabetes control. 

The All of Us Research Program, led by the National Institutes of Health (NIH), seeks to collect health data from one million participants, with a focus on underrepresented groups. The database includes survey responses, electronic health records, biospecimens, and linked health data across diverse racial, ethnic, and socioeconomic backgrounds. As of 2024, over 413,000 participants have enrolled, making it an ideal resource for studying disparities in diabetes care and treatment adherence. 

This study aims to leverage the All of Us dataset to examine whether specific aspects of patient-provider relationships - such as perceived respect, shared decision-making, and provider engagement - are associated with diabetes management. By identifying factors linked to poorly managed diabetes (PM DM), findings from this study may inform strategies to improve provider communication and patient adherence in diabetes care. 

This project was previously presented as a poster at the Mississippi Public Health Association Annual Conference on November 15, 2024, and the abstract was printed in the December 2024 edition of the Future DO Magazine. 

## Materials and methods

**Figure 1:**
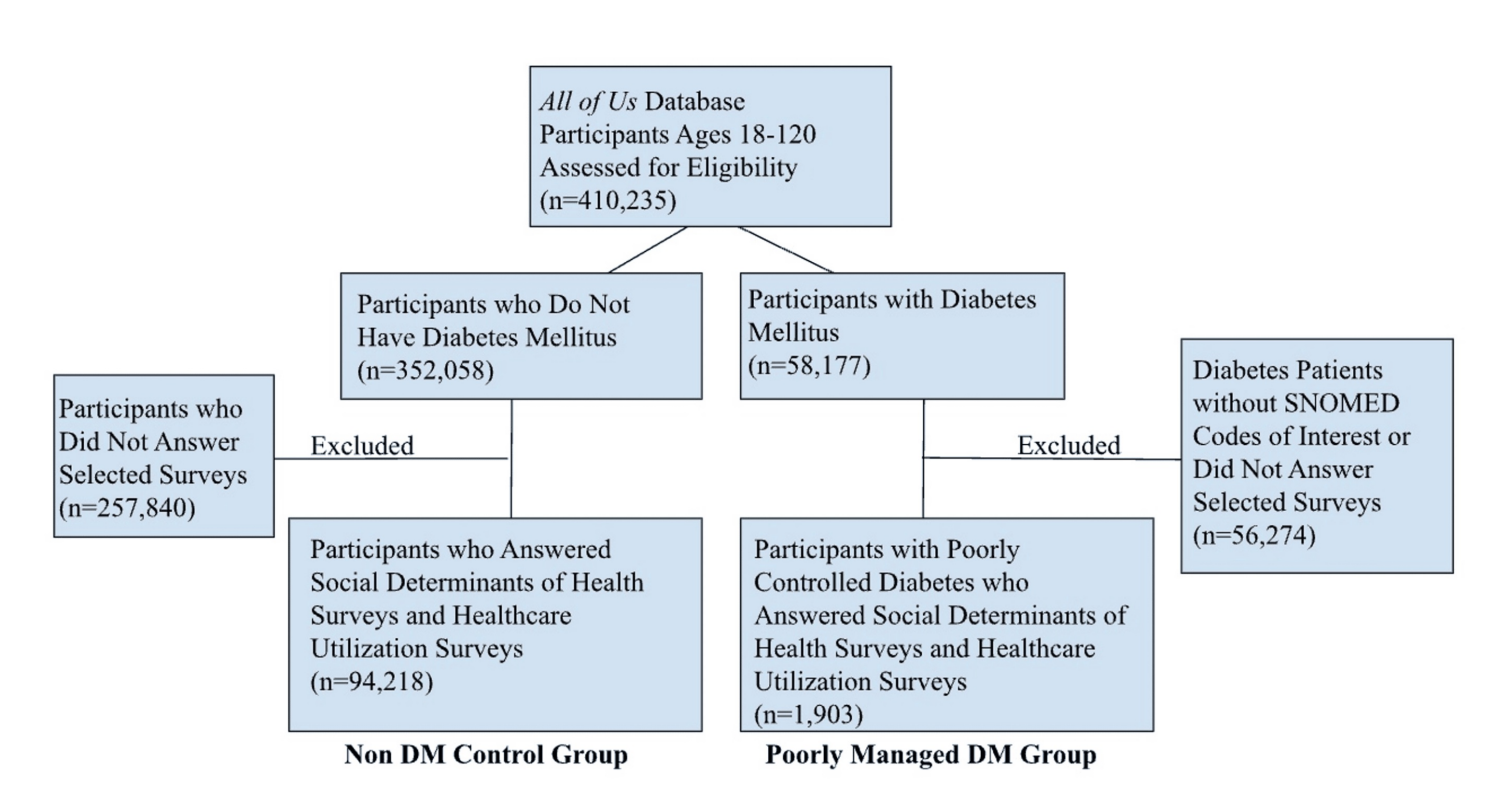
Consort diagram displaying the selection of participants for each cohort DM - diabetes mellitus, SNOMED - systematized nomenclature of medicine

This study employs a retrospective cross-sectional design using publicly available data from the All of Us Research Program (AoU). The AoU dataset was selected because it allows for a large-scale, representative analysis of patient-provider relationships and chronic disease management. Participants were divided into two cohorts (Figure 1). The first cohort, PM DM, included participants with systematized nomenclature of medicine (SNOMED) codes 268519009, 443694000, 444073006, and 45106100012, indicating uncontrolled diabetes. The second cohort of those without diabetes mellitus (No DM) included participants with no SNOMED codes indicating a diabetes diagnosis.

To evaluate patient-provider interactions, five key survey questions from the Social Determinants of Health and Healthcare Access and Utilization surveys were analyzed (Table 1). These questions assessed perceived respect from healthcare providers, the frequency with which patients were asked for their medical opinions, the importance of provider similarity (such as shared race, religion, or language), nervousness about seeking medical care, and whether patients felt their provider viewed them as less intelligent. 

**Table 1:** Selected survey questions from AoU database and possible answer choices AoU - All of Us Research Program

Survey questions	Answer choices
How often were you treated with less respect than other people when you go to a doctor’s office or other healthcare provider?	1= Always, 2= Most of the time, 3= Sometimes, 4= Rarely, 5= Never
How often did your doctors or health care providers ask for your opinions or beliefs about your medical care or treatment? For example, what kind of tests, procedures, or medications you prefer. Would you say…	1= Always, 2= Most of the time, 3= Some of the time, 4= None of the time
The following questions are about your experiences with doctors and other health care providers in the past year. Some people think it is helpful if their providers are from the same background that they are – like in terms of race or religion or native language –because they think their doctors will better understand what they’re experiencing or going through. How important is it to you that your doctors or health care providers understand or are similar to you in any of these ways? Would you say…	1= Very important, 2= Somewhat important, 3= Slightly important, 4= Not important
There are many reasons people delay getting medical care. Have you delayed getting care for any of the following reasons in the PAST 12 MONTHS? You were nervous about seeing a health care provider...	0= No, 1= Yes
The next statements describe how others may treat you. How often do any of these happen to you when you go to a doctor’s office or other health care provider? A doctor or nurse acts if he or she thinks you are not smart.	1= Always, 2= Most of the time, 3= Sometimes, 4= Rarely, 5= Never

Responses to these questions were collected using a Likert scale and were subsequently dichotomized for statistical analysis. Participants who reported feeling "always" or "most of the time" disrespected by their provider were categorized under high perceived disrespect, whereas all other responses were classified as low perceived disrespect. Similarly, responses regarding patient involvement in decision-making were grouped into high involvement (for those who answered "always" or "most of the time") and low involvement (for all other responses). Finally, the question regarding nervousness about seeing a provider was analyzed as a binary variable (yes/no). By structuring the data in this way, the study aimed to identify significant patterns in patient-provider relationships and their potential impact on diabetes management. 

To be included in the analysis, participants had to answer the five survey questions and be 18 years or older at the time of the survey response. 

Demographic variables, including race, ethnicity, and sex at birth, were analyzed for frequency and percent across categories. To identify associations between patient-provider interactions and poorly managed diabetes (PM DM) status, chi-square tests were performed to assess bivariate relationships. Chi-square tests were used to examine bivariate associations between patient-provider interaction variables and PM DM status. To control for potential confounders and determine the most significant predictors of PM DM, backward stepwise binary logistic regression was utilized. Variables that met the significance threshold of p<0.10 in bivariate analysis were entered into the model, and only those that remained significant at p<0.05 were retained in the final analysis. Adjusted odds ratios (AORs) and 95% confidence intervals (CIs) were calculated to quantify the strength of associations between patient-provider interaction factors and diabetes management. All analyses were performed using SAS Analytics Pro version 9.4 (SAS Institute Inc., Cary, North Carolina), available through the All of Us Researcher Workbench. 

## Results

After applying exclusion criteria to remove participants with incomplete survey responses or missing SNOMED codes, a total of 94,218 participants were included in the No DM cohort, while 1903 participants comprised the PM DM cohort. Demographic characteristics for both groups are presented in Table 2. The majority of participants were White (n=1498, 88.38%) in the PM DM cohort, and in the No DM cohort (n=72405, 89.09%). Most of the PM DM cohort (n=1693, 92.36%) and the No DM (n=81,109, 91.77%) cohort identified as non-Hispanic. Additionally, a greater proportion of participants were female, with 1239 (66.79%) of the PM DM cohort and 59,360 (65.98%) of the No DM cohort identifying as such. 

**Table 2:** Demographics of participants in the poorly managed (PM DM) and no diabetes mellitus (No DM) cohorts

	Poorly managed (PM DM) n (%)	No diabetes mellitus (No DM) n (%)	Total n (%)
Ethnicity			
Non-Hispanic	1693 (92.36)	81,109 (91.77)	82,802 (91.78)
Hispanic	140 (7.64)	7275 (8.23)	7415 (8.22)
Race			
White	1498 (88.38)	72,405 (89.09)	73,903 (89.07)
Black/AA	140 (8.26)	5833 (7.18)	5973 (7.20)
Asian	50 (2.95)	2605 (3.21)	2655 (3.20)
Native Hawaiian/other Pacific Islander	Not reported	52 (0.06)	Not reported
Middle Eastern/North African	Not reported	378 (0.47)	Not reported
Sex at birth			
Female	1239 (66.79)	59,360 (65.98)	60,599 (65.99)
Male	616 (33.21)	30,612 (34.02)	31,228 (34.01)

Analysis of patient-reported experiences with healthcare providers revealed statistically significant differences in several key areas. All frequencies and percentages of answers to the selected survey questions can be found in Table 3. Participants were asked whether they felt they were treated with less respect than others when seeing their healthcare providers. While responses were similar across both groups, a slightly higher number of PM DM patients, 31 (1.68%), reported always feeling less respected, compared to 1077 (1.19%) in the No DM group. Additionally, 1058 (57.38%) PM DM patients reported never feeling like they were treated with less respect, compared to 52,911 (58.41%) in the No DM group. The difference in responses between the groups was statistically significant (p=0.0005). 

**Table 3:** Frequency of participants' responses to each selected survey question PM DM - poorly managed diabetes mellitus, No DM - no diabetes mellitus

	PM DM n (%)	No DM n (%)	Total n (%)
How often were you treated with less respect than other people when you went to a doctor’s office or other healthcare provider? (p=0.0005)			
Always	31 (1.68)	1077 (1.19)	1108 (1.20)
Most of the time	23 (1.25)	905 (1.00)	928 (1.00)
Sometimes	230 (12.47)	8992 (9.93)	9222 (9.98)
Rarely	502 (27.22)	26701 29.48)	27203 (29.43)
Never	1058 (57.38)	52,911 (58.41)	53,969 (58.39)
How often did your doctors or health care providers ask for your opinions or beliefs about your medical care or treatment? For example, what kind of tests, procedures, or medications you prefer. Would you say… (p<0.0001)			
Always	600 (32.73)	21,967 (25.79)	22,567 (25.93)
Most of the time	595 (32.46)	27,675 (32.49)	28,270 (32.48)
Some of the time	420 (22.91)	24,612 (28.89)	25,032 (28.76)
Never	218 (11.89)	10,938 (12.84)	11,156 (12.82)
The following questions are about your experiences with doctors and other health care providers in the past year. Some people think it is helpful if their providers are from the same background that they are – like in terms of race or religion or native language –because they think their doctors will better understand what they’re experiencing or going through. How important is it to you that your doctors or health care providers understand or are similar to you in any of these ways? Would you say… (p=0.8840)			
Very important	497 (27.41)	25,161 (28.20)	25,658 (28.19)
Somewhat important	563 (31.05)	27,433 (30.75)	27,996 (30.76)
Slightly important	349 (19.25)	16,756 (18.78)	17,105 (18.79)
Not important	404 (22.28)	19,866 (22.27)	20,270 (22.27)
There are many reasons people delay getting medical care. Have you delayed getting care for any of the following reasons in the PAST 12 MONTHS? You were nervous about seeing a health care provider...(p<0.0001)			
No	1597 (90.12)	75,995 (86.72)	77,592 (86.79)
Yes	175 (9.88)	11,636 (13.28)	11,811 (13.21)
The next statements describe how others may treat you. How often do any of these happen to you when you go to a doctor’s office or other health care provider? A doctor or nurse acts if he or she thinks you are not smart. (p=0.0917)			
Always	28 (1.54)	1210 (1.35)	1238 (1.35)
Most of the time	21 (1.16)	1503 (1.68)	1524 (1.67)
Sometimes	185 (10.20)	9363 (10.45)	9548 (10.44)
Rarely	374 (20.63)	20,194 (22.53)	20,568 (22.49)
Never	1205 (66.46)	57,370 (64.00)	58,575 (64.05)

When asked how frequently the healthcare providers sought their opinions or beliefs about their medical care or treatment, 600 (32.73%) PM DM patients were significantly more likely to report always being asked, compared to 21,967 (25.79%) No DM patients (p<0.0001). Similarly, 595 (32.46%) PM DM patients stated they were asked for input most of the time, compared to 27,675 (32.49%) in the No DM group. 

Participants were also asked how important it was for them to have a healthcare provider who shared their race, religion, or language background. Responses were consistent across both groups, with 563 (31.05%) PM DM patients and 27,433 (30.75%) No DM patients reporting that this was 'somewhat important'. There was also no significant difference between the two groups when analyzing the percentage reporting that similarity was 'very important', with 497 (27.41%) in the PM DM cohort, and 25,161 (28.20%) in the No DM cohort (p=0.8840). 

A significant difference emerged when participants were asked whether they had delayed seeking care in the last 12 months due to being nervous about talking to their provider. While 1597 (90.12%) PM DM participants reported not delaying care due to nervousness, 75,995 (86.72%) No DM participants reported the same. This difference was statistically significant (p<0.0001). 

When asked whether they felt as though the healthcare provider acted as if they thought the patient was not smart, responses were similar across both groups. The majority of participants in both the PM DM (n= 1205, 66.46%) and No DM (n=57,370, 64.0%) cohorts reported never feeling this way. There was no statistically significant difference between the two groups (p=0.0917). 

**Figure 2:**
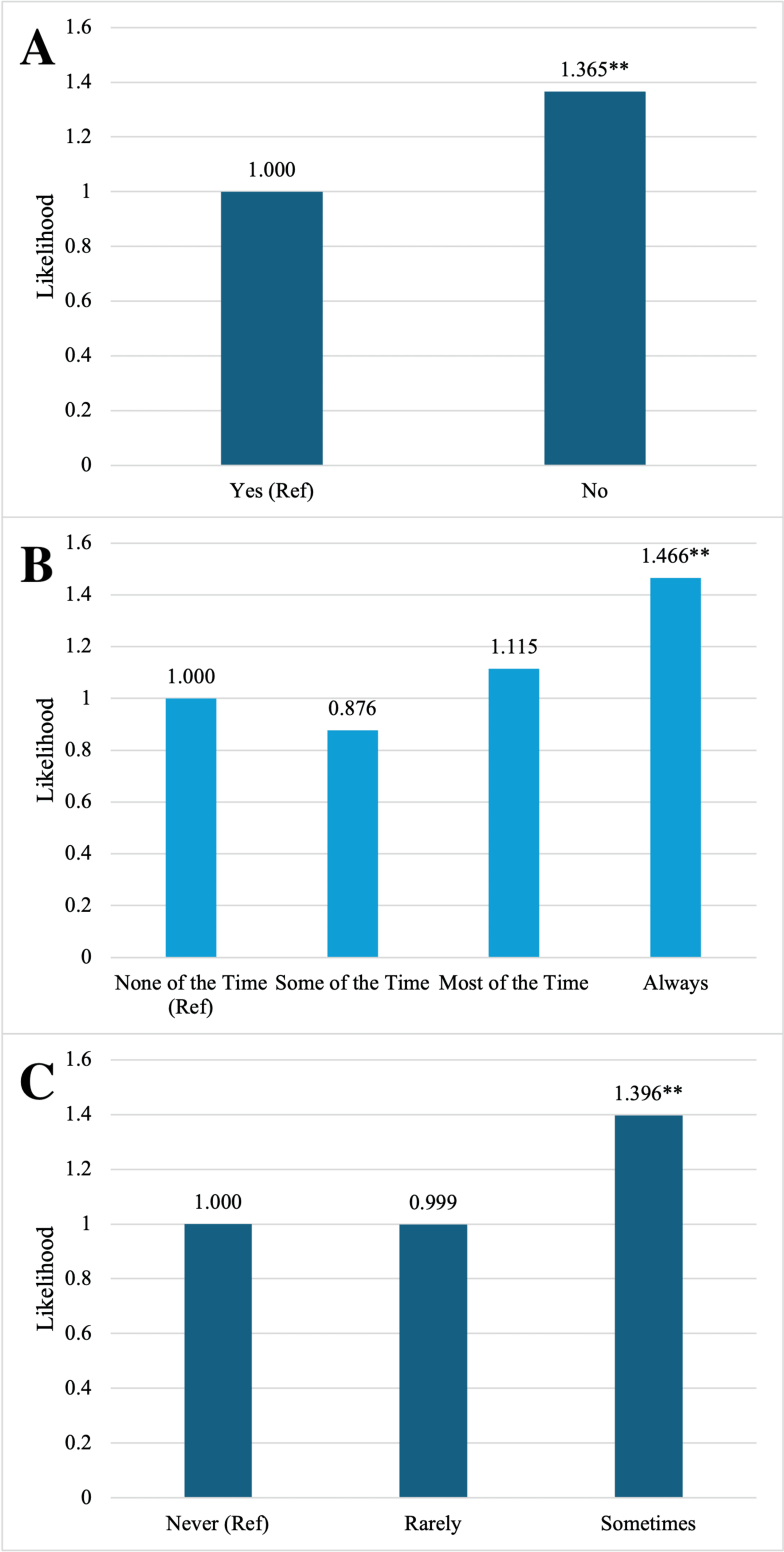
A) Multivariate analysis of the PM DM cohort to be nervous to see a provider, B) Multivariate analysis of PM DM cohort being asked their opinion in their care, C) Multivariate analysis of PM DM cohort to feel less respect than others at the doctor's office A) *When adjusted for opinion and respect **Significant Results. Independent variables were plotted against the reference (Ref) value of Exp (B)=1.0; B) *When adjusted for nervous and respect **Significant Results. Independent variables were plotted against the reference (Ref) value of Exp (B)=1.0; C) *When adjusted for nervous and opinion **Significant Results. Independent variables were plotted against the reference (Ref) value of Exp (B)=1.0. PM DM - poorly managed diabetes mellitus

Three key patient-provider interaction variables remained statistically significant in the adjusted multivariable model. Individuals who reported that they sometimes felt as though they were treated with less respect than others by their physician were 1.396 times more likely (95%CI: 1.189, 1.639) to have poorly managed diabetes than those who never felt as though they were treated with less respect. This association remained significant after adjusting for patient nervousness and ability to voice opinions (Figure 2A). 

Individuals who reported that they could always voice their opinions about their healthcare were 1.466 times more likely (95% CI: 1.235, 1.740) to have uncontrolled diabetes compared to those who were never able to voice their opinions. These results were adjusted for patient nervousness and perceived respect (Figure 2B). 

Individuals who reported that they were not nervous to see a provider were 1.365 times more likely (95%CI: 1.152, 1.618) to have poorly managed diabetes compared to those who reported being nervous. These results were adjusted for perceived respect and the ability to voice opinions (Figure 2C). 

These findings suggest that while feeling disrespected by a provider was associated with poorer diabetes management, higher levels of patient involvement and comfort in healthcare settings were also unexpectedly associated with poorer diabetes outcomes. Further research is needed to explore the complex dynamics underlying these associations. 

## Discussion

The physician-patient relationship is a well-established determinant of health outcomes, with studies consistently highlighting the importance of effective communication, mutual respect, and shared decision-making in fostering adherence to medical recommendations. A strong relationship is often built on shared values, cultural similarities, and open dialogue, all of which contribute to improved compliance with treatment regimens [17]. Prior studies have shown that certain groups of patients may feel stigmatized by their provider [18], which can lead to a breakdown of the relationship between physician and patient. Other studies have shown that physicians who rarely ask questions, do not make eye contact with their patients, or fail to spend adequate time with them often result in a lack of motivation on the patient's part to maintain their treatment or therapeutic regimen [19-22]. However, research also indicates that perceived stigma, poor communication, and physician disengagement can erode trust, leading to diminished adherence and worse health outcomes. Given these findings, the present study sought to examine how patient perceptions of respect, involvement in decision-making, and comfort with providers relate to diabetes management. Interestingly, while the current study confirmed prior research showing that perceived disrespect from providers was associated with poorer diabetes management, it also produced unexpected results regarding patient comfort and involvement in care. Specifically, patients in the PM DM cohort reported being asked for their opinions on care more frequently than those in the No DM cohort. Additionally, those who were less nervous about seeing a provider were more likely to have poorly managed diabetes. These findings challenge traditional assumptions that increased patient engagement and comfort lead to better adherence and warrant further exploration into the nuances of patient-provider dynamics in chronic disease management. 

Our study revealed that patients who sometimes felt as though they were treated with less respect than others when being seen by a physician were 1.396 times more likely to have poorly managed diabetes than those who never felt disrespected. This finding is in agreement with a previous study indicating that patients who perceive disrespect are less likely to adhere to medical recommendations [23]. Previous research suggests that disrespect may manifest in various ways, including dismissive attitudes, rushed appointments, or lack of active listening, which can discourage patients from following prescribed treatment regimens. 

Non-compliance is not always a choice, however. Many patients struggle with adherence due to structural barriers such as financial constraints, lack of transportation, or workplace inflexibility [24]. Studies have shown that patients who are unable to abide by their physician's instructions often feel blamed or judged by their physician, further straining the patient-provider relationship [16]. Due to these extrinsic factors that may be at play in any patient's life at any given time, physicians must consider that non-compliance may stem from external challenges rather than intentional defiance. 

Another consideration is that diabetic patients visit a doctor's office approximately three times more often than the general population [25]. This is helpful in explaining our study's finding that individuals who were not nervous to see their provider were 1.365 times more likely to have poorly managed diabetes than those who reported being nervous. Because diabetics see a physician so frequently, these individuals may be more at ease in the healthcare setting due to familiarity with their provider. However, Papaspurou et al. named a plethora of anxieties that diabetics suffer from [26]. Further, this study also found that making the patient aware of changes in their care may alleviate some of their fears and anxieties that plague their everyday lives. This suggests that participants with poorly managed diabetes may be properly and adequately informed about their therapeutic regimen, causing a decline in overall nervousness when seeing a provider. 

It has also been found that when a patient takes an active role in their care, it can lead to better outcomes [5,27]. This is in opposition to this study's finding that patients who were always able to voice their opinions about their healthcare were 1.466 times more likely to have a case of uncontrolled diabetes than those who were never able to voice their opinions. It would seem more likely that individuals who have poorly managed diabetes would be asked their opinion less often, and as a result, be less compliant with their treatment. After all, in order to maintain a healthy relationship between a patient and their provider, it is crucial to have patients involved in the construction of their treatment regimen [28,29]. However, because of studies like Heisler et. al., it is possible that physicians have learned to let the patient take a larger role in their medical decision making [27]. This suggests that the dynamics of patient-provider interactions may have evolved, with physicians increasingly allowing patients a greater role in decision-making, which might contribute to unexpected outcomes in disease management. 

Although race-concordance theory suggests that patients often report higher satisfaction and better adherence when they share racial, ethnic, or linguistic similarities with their provider, our study found no significant difference in the importance of demographic similarity between the PM DM and No DM cohorts. This suggests that factors beyond race and ethnicity may play a more influential role in diabetes management [30,31]. Studies regarding race-concordance theory have stated that when a patient and provider share a kindred identity, a patient may have better access to and satisfaction with their care. Traylor et al. even went on to show that this concordance can influence adherence to treatment among African Americans and Spanish-speaking patients [32]. This highlights the crucial role of cultural competence in healthcare, emphasizing that shared identity between patient and provider can significantly impact health outcomes and treatment adherence. 

The difference in responses between the non-diabetic participants and the poorly managed diabetics cannot be explained by a demographic disparity, as the uncontrolled diabetic cohort and non-diabetic cohort were not significantly different with regard to race, sex at birth, or ethnicity. This aligns with previous studies that have stated no notable impacts have been linked to racially concordant physician-patient relationships [33,34]. However, while the cohorts were not different from each other, the overall representation of the cohort differed from the United States' general population, which poses the first limitation of our study. Both cohorts were predominantly female. The white demographic of the current study was 89.09% compared to a 75.30% white population in the United States [35]. Finally, the ethnicity for our cohort was 91.78% non-Hispanic, whereas the country's population is 80.50% non-Hispanic. The racial breakdown of the cohorts utilized in this study was a bit of a surprise, as All of Us Database seeks to bring more representation to minority groups [36]. It also must be considered that over 13,000 members of our study failed to self-identify their race. These demographic discrepancies highlight just a few of the limitations of our study and underscore the need for more representative sampling to ensure the findings are generalizable to the broader US population. 

Other limitations of our study involve the small number of participants in the uncontrolled diabetic group, as compared to the non-diabetic cohort. This smaller sample size might not fully represent the experiences and feelings of poorly controlled diabetics. Further, another constraint is that patients were selected for participation depending on their diagnosis at a certain snapshot in time. It cannot be determined whether any of the uncontrolled diabetics eventually became well-managed or if any non-diabetics later developed diabetes. Ultimately, these limitations underscore the importance of further research with larger, more diverse populations and longitudinal data to gain a more comprehensive understanding of the issues at hand. 

A long-lasting implication of the current study is that providers should aim to display respect to every patient, but should pay special attention to their disposition towards non-compliant patients. Future studies should investigate the findings of a non-compliant patient being asked for their opinion in their care more frequently. It should be determined whether this is a result of other studies cited as a method to increase patient compliance, or if non-compliant patients are feeling overwhelmed by their options. Further research should continue to explore non-compliance and the patient-provider relationship in conditions beyond diabetes. Ultimately, addressing non-compliance requires a multi-faceted approach, looking not only at the patient-provider relationship but the external factors that influence each patient's ability and desire to adhere to treatment. 

Future research should aim to clarify these relationships across different populations and chronic conditions, examining how specific patient-provider interactions influence long-term health outcomes. Several limitations must be acknowledged in this study. The underrepresentation of certain demographic groups and the small sample size of patients with poorly managed diabetes may have influenced the findings. Additionally, the cross-sectional study design prevents causal conclusions about the relationship between patient perceptions and treatment adherence. Ultimately, addressing non-compliance requires a multifaceted approach, integrating relational factors, patient-centered communication, and systemic barriers to care. By advancing these insights, healthcare providers and policymakers can develop more effective interventions to improve chronic disease management and overall health outcomes.

## Conclusions

In conclusion, this study highlights the significant impact of the patient-provider relationship on treatment compliance, particularly for chronic conditions like diabetes. Findings from this study reveal a complex and somewhat paradoxical dynamic between patient-provider interactions and diabetes management. While patients with poorly managed diabetes reported feeling disrespected by their providers, they were also less nervous about medical visits and more frequently asked for their opinions on treatment decisions. These results challenge conventional assumptions that greater patient involvement and comfort necessarily lead to better adherence. Additionally, this study underscores the importance of culturally competent care in fostering strong patient-provider relationships. Although racial and ethnic similarity between patients and providers has been shown to enhance trust and satisfaction, our study found no significant differences in treatment compliance based on demographic similarity. This suggests that factors beyond shared identity, such as provider communication style and systemic barriers, may play a more crucial role in adherence.

Future research should aim to clarify these relationships across different populations and chronic conditions, examining how specific patient-provider interactions influence long-term health outcomes. Several limitations must be acknowledged in this study. The underrepresentation of certain demographic groups and the small sample size of patients with poorly managed diabetes may have influenced the findings. Additionally, the cross-sectional study design prevents causal conclusions about the relationship between patient perceptions and treatment adherence. Ultimately, addressing non-compliance requires a multifaceted approach, integrating relational factors, patient-centered communication, and systemic barriers to care. By advancing these insights, healthcare providers and policymakers can develop more effective interventions to improve chronic disease management and overall health outcomes.
